# Early-Life Swine Inflammation and Necrosis Syndrome Is Associated with Later Tail Integrity and Systemic Hematological Changes in Organically Raised Pigs

**DOI:** 10.3390/ani16131962

**Published:** 2026-06-25

**Authors:** Karien Koenders-van Gog, Esther Krooshoop, Thomas Wijnands, Gerald Reiner

**Affiliations:** 1 Merefelt Livestock Diagnostics, Lintjeshof Veterinary Practice, LH Vet Group, 6031 RK Nederweert, The Netherlands; k.koenders@lintjeshof.com; 2 Lintjeshof Veterinary Practice, LH Vet Group, 6031 RK Nederweert, The Netherlandst.wijnands@lintjeshof.com (T.W.); 3 Clinic for Swine—Herd Health Management and Molecular Diagnostics, Justus-Liebig-University Giessen, 35392 Giessen, Germany

**Keywords:** tail biting, welfare, swine, organic production

## Abstract

Swine Inflammation and Necrosis Syndrome (SINS) is a widespread condition in pigs and has been proposed as an early-life animal-based measure (ABM) for assessing health and welfare. However, its prognostic value for later-life outcomes under commercial conditions remains poorly understood. This study investigated the prevalence, progression, and predictive relevance of SINS in two organic pig farms in the Netherlands. Clinical SINS signs were assessed in suckling and weaned piglets and related to hematological parameters at weaning (35 weaned piglets) as well as tail integrity at slaughter. SINS lesions were highly prevalent in suckling piglets (approximately 80%) but markedly decreased after weaning. Lesion prevalence and severity differed substantially between farms and showed clear age-dependent patterns, peaking between days 3 and 5 of life. Higher SINS scores in suckling piglets were associated with systemic hematological alterations at weaning, including increased monocyte proportions, reduced platelet counts, and altered red blood cell indices. Importantly, early-life SINS was significantly associated with later tail integrity. Pigs with higher SINS scores showed a lower probability of intact tails at slaughter and subsequently a higher prevalence of tail lesions. These findings suggest that SINS may have potential as an early-life indicator of later tail outcomes; however, this hypothesis requires validation in larger studies involving a greater number of farms and production systems.

## Simple Summary

Swine Inflammation and Necrosis Syndrome (SINS) is a common condition affecting pigs of different ages and is characterized by inflammatory lesions in body regions such as the tail, ears, and claws. This study investigated whether inflammatory signs observed in young piglets can help predict later tail health in pigs raised under organic farming conditions. The study was initiated with 361 pigs with intact tails from two organic farms in the Netherlands. Clinical signs of SINS were recorded in suckling and weaned piglets, and tail condition was assessed later during finishing and at slaughter in the animals available for follow-up. No tail biting and only mild inflammatory tail lesions in a small proportion of piglets occurred. Piglets showing more pronounced SINS signs early in life were less likely to have fully intact tails at slaughter. In addition, blood analyses at weaning revealed that pigs with higher SINS scores showed changes in immune and red blood cell parameters, indicating a systemic inflammatory response. These findings suggest that SINS can serve as an early animal-based indicator to identify pigs at increased risk of later tail problems. Early detection may allow farmers to implement targeted preventive measures and improve animal welfare in pig production systems.

## 1. Introduction

Tail lesions in pigs represent a major animal welfare concern and are of central relevance for both producers and consumers [1,2,3,4,5]. Increasing regulatory requirements and consumer expectations demand the rearing and marketing of pigs with intact tails [6,7]. However, practical experience indicates that the maintenance of completely uninjured tails cannot be guaranteed, as multifactorial influences—including environmental stress, management practices, and early local inflammatory processes—substantially affect tail health [8].

To address this challenge, animal-based measures (ABMs) have been proposed as particularly suitable tools for assessing animal welfare directly at the individual level. In this context, Koenders-van Gog et al. [9,10] suggested that Swine Inflammation and Necrosis Syndrome (SINS) may serve as an early clinical indicator in piglets. Animals exhibiting SINS lesions at an early age were shown to have an increased risk of developing tail lesions later in life, indicating that SINS could function as an early, animal-based marker for individual risk assessment and targeted preventive interventions (“point of care”).

The use of ABMs such as SINS may therefore enable a more proactive management of animal welfare and husbandry practices. In contrast to environmental or management indicators, ABMs directly reflect the animals’ clinical condition and may thus provide practical tools for early risk assessment of tail health [9].

Swine Inflammation and Necrosis Syndrome is a widespread condition characterized by inflammatory alterations affecting multiple body regions, including the tail, ears, teats, coronary bands, and heels [11]. The syndrome typically begins with mild clinical signs such as bristle loss, redness, and swelling, but may progress to more severe manifestations including exudation, necrosis, and hemorrhages [12]. Consequently, SINS can be regarded as an early animal-based measure that allows interventions to be initiated at the onset of clinical signs and may stimulate the implementation of preventive management strategies.

SINS has been hypothesised to arise from systemic inflammatory processes involving microbe-associated molecular patterns (MAMPs), particularly lipopolysaccharides (LPS) [13,14,15,16]. Gastrointestinal dysfunction, dysbiosis, impaired intestinal barrier integrity, and mycotoxins such as deoxynivalenol (DON) have been discussed as potential contributors to endotoxin translocation and inflammatory activation [17,18,19,20,21,22,23,24,25,26,27,28,29,30,31]. Excessive exposure to MAMPs may induce inflammatory and metabolic responses involving the liver, oxidative stress, endothelial activation, and microvascular dysfunction [32,33,34,35,36,37,38]. Histopathological, transcriptomic, metabolomic, and genetic studies in SINS-affected piglets provide support for the involvement of inflammatory, metabolic, and vascular pathways in the syndrome [39,40,41,42,43]. However, these mechanisms were not investigated in the present study and are mentioned only as potential explanatory concepts.

SINS has been described in pig farms across several countries, including Germany, the Netherlands, France, Italy, and Brazil [9,10,20,44]. Considerable variation in prevalence and severity has been reported both within and between studies [10,44]. For example, a study in Northern Italy on slower-growing, less lean pigs used for Parma ham production reported markedly lower levels of SINS compared with previous findings, indicating a potential influence of genetic and production system factors [9]. In addition, improvements in water hygiene and dietary fiber supply have been shown to significantly reduce SINS prevalence across production stages [39].

Importantly, even low-grade manifestations of SINS have been associated with relevant production and health traits [9,12]. Such associations may be particularly relevant in organic production systems, where variability in management and environmental conditions may promote distinct patterns of early clinical expression.

Another key aspect of SINS as an ABM is the association between clinical manifestations and systemic responses. Previous studies have demonstrated links between SINS scores and alterations in clinical-chemical and hematological parameters, highlighting the systemic nature of the syndrome [9,41,42,43]. Notably, Loewenstein et al. [41] reported that pronounced clinical signs in suckling piglets were associated with measurable systemic changes only at the weaning stage, suggesting that visible lesions at acral sites precede systemic physiological adaptations.

Whereas previous SINS studies from our group primarily focused on clinical characterization, pathophysiology, genetic associations, or cross-sectional prevalence, the present study followed individually identified pigs from birth through weaning, finishing, and slaughter and linked early-life SINS signs with later hematological and tail-related outcomes under commercial organic farming conditions.

Against this background, the present study aimed to evaluate whether SINS in piglets from organic farming systems can serve as a prognostic indicator for later tail health. Furthermore, the study investigated whether associations exist between clinical findings in suckling and weaned piglets and hematological parameters measured at weaning, particularly under conditions of low-grade disease expression, and whether these relationships may contribute to improved diagnostic and prognostic approaches for tail health in finishing pigs.

## 2. Materials and Methods

### 2.1. Study Design

The study was conducted on two closed pig farms in the Netherlands operating according to the specifications of certified organic farming (SKAL) between March and December 2025. Farms were selected based on the following criteria: production of pigs with intact tails, use of digital ear tags (LeeO; LeeO B.V., Deventer, The Netherlands), veterinary supervision by Lintjeshof Veterinary Practice, and delivery of slaughter pigs to Vion slaughterhouse (Vion Apeldoorn B.V., Apeldoorn, The Netherlands). Both institutions were partners in the EU-funded project TAILSCAN.

Ethical review and approval were waived for this study, as all procedures were conducted within the framework of routine herd health management. Blood sampling and clinical examinations were performed for veterinary diagnostic purposes, and data were subsequently used for scientific evaluation. No additional procedures or interventions were carried out beyond standard farm management.

**Figure 1:**
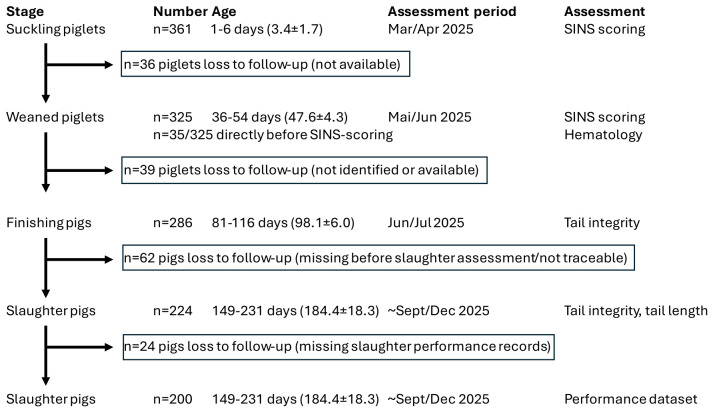
Study design and animal flow throughout the longitudinal study. Clinical signs of swine inflammation and necrosis syndrome (SINS) were assessed in suckling and weaned piglets. Tail integrity was assessed in finishing pigs and at slaughter. Numbers of animals available and losses during follow-up are shown for each production stage. Losses occurred under commercial farm conditions and included mortality, inability to relocate individual animals during subsequent assessments, slaughter before scheduled recording, and unsuccessful linkage between farm and slaughterhouse identification systems.

Swine inflammation and necrosis syndrome (SINS) was assessed in suckling piglets at 1–6 days of age and in weaned piglets at the time of weaning. Under organic farming conditions, piglets were weaned at a mean age of 36–54 days; the mean age at weaning was 46.6 ± 4.2 days on Farm 1 and 50.8 ± 2.5 days on Farm 2. Tail length and tail integrity were further assessed at the beginning of the finishing period (98.1 ± 6.0 days of age) and at slaughter (184.4 ± 18.3 days of age) (Figure 1).

For statistical analyses, age at weaning was recoded into the following clusters: D40 (days 36 and 40, *n* = 30), D45 (days 44–46, *n* = 69), D48 (days 47–49, *n* = 131), and D52 (days 50–54, *n* = 95).

SINS data were available for 361 suckling piglets (Farm 1: 247; Farm 2: 114) and 325 weaned piglets (Farm 1: 247; Farm 2: 78). Hematological data were available from 35 out of the 325 weaned piglets (Farm 1: 18; Farm 2: 17). Tail integrity and tail length data were available from 286 pigs at the beginning of the finishing period (Farm 1: 224; Farm 2: 62) and from 224 pigs at slaughter (Farm 1: 183; Farm 2: 41). Slaughter performance data were available from 200 pigs (Farm 1: 169; Farm 2: 31).

The study had a longitudinal design, with suckling/weaning, finishing, and slaughter data collected from the same individuals wherever possible. Complete tail-related data from the suckling period to slaughter were available for 224 animals; the remaining animals were either lost during follow-up or could not be reliably tracked (Figure 1).

### 2.2. Farm Characteristics

The farms were selected prospectively according to predefined inclusion criteria. Eligible farms had to operate under certified organic production conditions (SKAL certification), keep pigs with intact tails, use LeeO digital ear tags to allow reliable individual animal identification throughout the production cycle, and operate as closed herds including breeding, nursery, and finishing units on the same farm. In addition, farms had to be clients of Lintjeshof Veterinary Practice and partners in the TAILSCAN project, be willing to participate while maintaining normal commercial production, and deliver slaughter pigs to the Vion slaughterhouse in Apeldoorn, where the TAILSCAN camera system (Farm4Trade, Bologna, Italy) was installed. Because only a limited number of Dutch organic farms fulfilled all of these criteria, the final study population comprised two farms.

The main characteristics of the two study farms are summarized in Table 1. Detailed descriptions of both farms are provided below. No dietary interventions were introduced for the purpose of this study. All feed components, including the absence of mycotoxin binders in the concentrate feed, reflected routine farm management practices. Farm 1 was a closed herd comprising *n* = 99 sows of Topigs L and T genetics and using PIC (Pig improvement company, Isernhagen, Germany) boars for artificial insemination. The farm was located in the eastern Netherlands in an area of moderate swine density and was considered PRRS (Porcine Reproductive and Respiratory Syndrome)-unstable based on long-term serological and PCR monitoring. Gilts, sows, and piglets were vaccinated against PRRS using a modified live vaccine. Piglets were additionally vaccinated against Porcine Circovirus type 2 (PCV2), *Mesomycoplasma (M.) hyopneumoniae*, porcine intestinal adenomatosis (PIA), and *Brachyspira pilosicoli* (autogenous vaccine). At the beginning of the finishing period, pigs also received vaccinations against *M. hyopneumoniae* and *Actinobacillus pleuropneumoniae* (APP).

Gestating sows were housed in straw-bedded group pens and fed via electronic feeding stations. Animals received commercially supplied pelleted feed and silage. Sows had access to an outdoor area and, during summer, to pasture. Water was provided via easily accessible drinkers delivering > 2 L/min, with one drinker per 10 sows.

Lactating sows were kept in free-range farrowing pens with a combination of concrete flooring covered with straw and metal slats. The piglet nest was located in a slightly lowered corner of the pen. Sows and piglets shared open drinkers and had access to an outdoor area. Indoor climatic conditions were comparable to outdoor conditions.

The nursery consisted of partially covered pens with straw-bedded concrete flooring and metal slats, housing 20 pigs per pen. Each pen was equipped with five bowl drinkers and eight feeding places. Piglets were mixed at weaning and moved directly to the nursery. Animals had access to an outdoor area and were fed pelleted feed. Health issues observed at the time of assessment included porcine intestinal adenomatosis. Nursery mortality ranged from 1.5% to 3.0%.

Finishers were housed in groups of 60 pigs on straw-bedded concrete floors with access to an outdoor area. Animals received pelleted feed supplemented with silage, and water was provided via drinkers at a ratio of one per 10 pigs. Pigs were treated individually only when necessary.

Farm 2 was a closed herd comprising *n* = 62 sows of Topigs L and Z genetics and using Tempo-growth boars for artificial insemination. The farm was located in the southern Netherlands in an area of high swine density and was considered PRRS-stable based on long-term serological and PCR monitoring. Gilts and sows were vaccinated against PRRS using a modified live vaccine. Piglets were vaccinated against PCV2, *Mesomycoplasma hyopneumoniae*, and porcine intestinal adenomatosis (PIA).

Gestating sows were housed in straw-bedded group pens and fed outdoors twice daily in open feeding stalls. Animals received commercially supplied pelleted feed. Sows had access to an outdoor area and, during summer, to pasture. Water was provided via easily accessible drinkers delivering > 2 L/min, with one drinker per 10 sows.

Lactating sows were kept in free-range farrowing pens with concrete flooring covered with straw. The piglet nest was located at the front of the pen and was covered. Sows and piglets shared open drinkers and had access to an outdoor area. Indoor climatic conditions were comparable to outdoor conditions. After weaning, piglets remained in the farrowing unit for approximately two weeks.

The nursery consisted of straw-bedded concrete floors, housing 10–15 pigs per pen. Each pen was equipped with four nipple drinkers and four feeding places. Piglets had access to an outdoor area and were fed pelleted feed. No health issues were observed at the time of assessment. Nursery mortality was <1.5%.

Finishers were housed in groups of 40 pigs on straw-bedded concrete floors combined with metal slats, with access to an outdoor area. Animals were fed pelleted feed, and water was provided via nipple drinkers at a ratio of one per five pigs.

Pigs were treated individually only when necessary.

### 2.3. Animal Assessment

Pigs originating from the two organic herds were individually monitored from the first week of life until slaughter. Animals were identified using digital ear tags (LeeO). Clinical assessments were performed at three production stages: during the first week of life, at weaning, and during the first weeks after transfer to the finishing unit. At slaughter, digital photographs of the tails were taken and evaluated. Blood samples were collected immediately after scoring at weaning as part of routine veterinary disease monitoring; these samples were additionally used for the present study.

**Figure 2:**
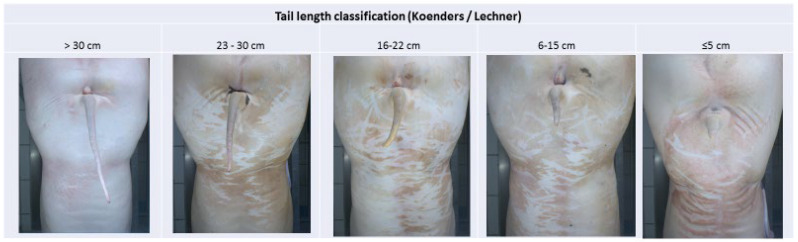
Tail length classification according to Koenders et al. [9].

**Figure 3:**
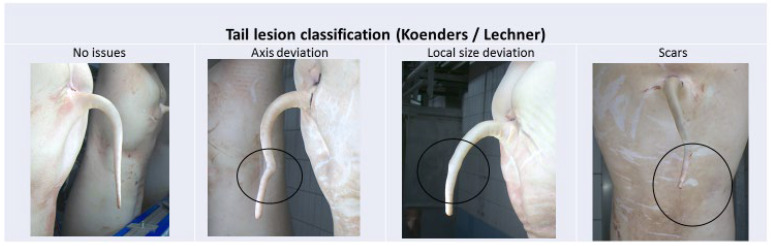
Tail lesion classification at slaughter according to Koenders and Lechner (see also Table 3). The circles in the figure highlight the respective changes.

Assessments in finishing pigs and slaughter pigs were performed by a single examiner. Assessments in suckling and weaned piglets were conducted jointly by two examiners using standardized scoring protocols (Table 2 and Table 3; Figure 2 and Figure 3). Both examiners assessed each animal simultaneously and agreed on a single consensus score at the time of examination. Therefore, no allocation of animals to different examiners occurred, minimizing potential inter-observer variation. Prior to data collection, both examiners were familiar with the scoring criteria and used the same standardized definitions and reference material throughout the study.

To minimize observer bias, results from previous or subsequent production stages were not available to the examiners at the time of assessment. Consequently, scoring at each production stage was performed independently of observations recorded at other stages of the study. According to [10,11], the following clinical parameters were assessed in neonatal and weaned pigs and recorded as present or absent (Table 2). At the beginning of the finishing period, only the tails were assessed, according to the scheme shown in Table 3.

### 2.4. Tail Assessment at the Slaughterhouse

Pigs were slaughtered at an age of approximately 6–7 months. At slaughter, three digital photographs of each individual tail were taken using the TAILSCAN camera system (Farm4Trade, Abruzzo, Italy). Photographs were obtained from standardized angles using three cameras.

According to Koenders-van Gog et al. [9], tail length was not measured as a continuous variable from the photographs. Instead, tails were visually classified into predefined length categories (>30 cm, 23–30 cm, 16–22 cm, 6–15 cm, and <5 cm) according to a standardized assessment protocol developed within the TAILSCAN project.

This approach was chosen because reliable automated measurement of undocked tail length from slaughterhouse photographs remains technically challenging and the AI-based assessment system developed within TAILSCAN had not yet completed validation at the time of the present study. The classification system was based on a previously validated photographic tail-length assessment approach developed within the German GenEthisch project. A dedicated training set was prepared by an experienced assessor involved in that project, and the assessor responsible for the present study received in-person training before evaluation commenced.

Three standardized photographs were obtained for each tail using a stereo-camera system installed at the slaughter line. Classification was performed using the central image, while the additional lateral views were used to support interpretation in cases of tail curvature or axis deviation. All assessments were conducted by a single trained assessor to ensure consistency across evaluations.

Tail integrity was evaluated visually using all three photographs of the same tail taken from different angles, according to the scoring scheme shown in Table 4 and illustrated in Figure 3 (see Koenders-van Gog et al. [9]).

### 2.5. Hematology

Blood samples were analyzed at Merefelt Livestock Diagnostics (Lintjeshof LH Vet Group, Nederweert, The Netherlands) using an automated veterinary hematology analyzer (ProCyte Dx, IDEXX Laboratories, Westbrook, ME, USA), based on fluorescence laser flow cytometry [45].

### 2.6. Statistical Analysis

Statistical analyses were performed using IBM SPSS Statistics version 29 (IBM, Munich, Germany).

Individual clinical signs (e.g., bristle loss, redness, swelling, exudation, necrosis, and bleeding) were initially recorded as binary variables (0 = absent, 1 = present). For each anatomical region, the individual signs were combined into body-part scores. For prevalence analyses, body-part scores were subsequently transformed into binary variables indicating whether the respective body region was affected (1) or unaffected (0).

The overall SINS score was calculated as: SINS score = tail base + tail tip + ear base + ear tip + coronary band + teats + heels, resulting in a theoretical score range from 0 to 7. The observed SINS score categories ranged from 0 to 5 and included 84, 92, 73, 63, 35, and 14 piglets, respectively.

For prevalence analyses, the overall SINS score was additionally converted into a binary variable (SINS absent/present). For association analyses, however, the original SINS score (0–5) was retained and used as an explanatory variable to assess relationships with hematological parameters, tail traits, and performance outcomes.

Tail-related findings in finishing and slaughter pigs were processed analogously. Individual tail lesions and abnormalities were analyzed as binary variables (0 = absent, 1 = present).

Binary outcome variables were analyzed using generalized linear mixed models (GLMM; GENLINMIXED) with a binomial distribution and logit link function. Fixed effects included farm, sex, and age, as well as the interactions farm × age and farm × sex. Random effects were sows nested within farm and examination day nested within farm. Sow nested within farm was included as a random effect to account for clustering of littermates. Examination day nested within farm was included as a random effect to account for potential batch-related variation. Due to the limited sample size and the pronounced differences between farms, the degrees of freedom did not allow inclusion of additional minor factors in the final models. Litter size and parity were evaluated in preliminary univariable and multivariable analyses; however, no significant effects were detected. As both variables were significantly correlated, they were not included in the final models. Where relevant, the original SINS score (0–5) was included as a metric covariate.

Prevalences are presented as standardized prevalences. Estimated marginal means derived from logistic mixed models systematically underestimated the observed overall prevalence because of the nonlinearity of the logit link function and the unbalanced data structure. Therefore, model-based prevalence estimates were reweighted and marginalized in a post hoc step. Numerically very small estimates corresponded to effective zero prevalences in groups without observed events.

Because marked farm effects and frequent interactions were observed, additional analyses were performed separately for each farm. In these farm-specific models, the fixed effect of farm was omitted, whereas sow and examination day were retained as random effects.

Although SINS data in suckling and weaned piglets were collected longitudinally at the individual animal level, these age groups were analyzed separately because they represent biologically distinct production stages with limited comparability.

Associations between hematological parameters and SINS were analyzed using generalized linear mixed models with hematological parameters as dependent variables and SINS score (0–5), sex, and age at assessment as fixed effects. Farm, sow, and examination day were included as random effects. Because no significant farm interactions were detected, hematological analyses were not stratified by farm.

Associations between calcaneal lesions and SINS traits were analyzed separately for each farm using binary generalized linear mixed models. Calcaneal inflammation (0/1) served as the dependent variable, whereas sex, age, and the respective SINS trait were included as fixed effects. Sow and examination day were included as random effects. Only statistically significant associations are reported.

Tail-related traits at slaughter were analyzed using generalized linear mixed models with farm and sex as fixed effects and sow nested within farm as well as examination day nested within farm as random effects. To investigate associations between tail traits and early-life SINS, the original SINS score (0–5) was included as an explanatory variable. Only statistically significant associations are reported.

Performance parameters were analyzed analogously using generalized linear mixed models.

Because only a few pigs at the beginning of the finishing period showed signs of disturbed tail integrity, associations at this stage were analyzed separately by farm using contingency table analyses. For sparse data, Fisher’s exact test was applied.

The study was designed as a pilot study with an exploratory and hypothesis-generating character. Therefore, no correction for multiple testing (e.g., Bonferroni adjustment) was applied in order to avoid overlooking potentially biologically relevant associations. Consequently, all results should be interpreted as exploratory.

## 3. Results

### 3.1. SINS in Suckling Piglets

The prevalence of SINS-associated lesions varied markedly depending on age (days of life), affected body region, and farm (Table 5 and Table S1). Overall, the highest prevalences were observed at the tail base, heels, and teat region, whereas lesions affecting the ears, coronary bands, and tail tip occurred less frequently. Nevertheless, SINS was detectable in approximately 80% of suckling piglets.

Over time, the prevalence of several lesion types increased markedly from the third day of life onwards. This increase was particularly pronounced for swelling, exudation, and necrotic changes at the tail base, as well as for swelling and bleeding in the heels. Although temporal patterns differed among lesion types, many lesions reached their highest prevalences between days 3 and 5 and subsequently remained stable or decreased slightly by day 6. Lesions observed during the first two days of life were generally mild and mainly consisted of redness or slight swelling (Table 5).

**Figure 4:**
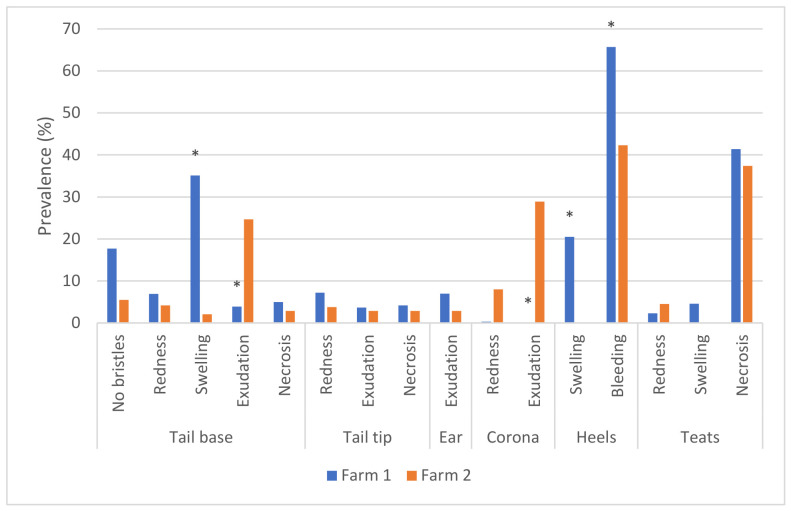
Effect of the farm on SINS signs in different body parts of suckling piglets, standardised by age and sex; *: the respective SINS signs are statistically significant.

Marked differences in both frequency and severity of SINS lesions were observed between the two farms (Table 5, Figure 4). Farm 1 showed substantially higher prevalences across numerous lesion types and body regions compared with Farm 2. This was particularly evident for bristle loss and tail base swelling on day 3, heel swelling on days 3 and 4, heel bleeding from day 4 onwards, teat necrosis on day 5, and ear exudation from day 6 (Figure S1). In contrast, several lesion types occurred only at low prevalence or were absent on Farm 2, although individual lesions such as coronary band exudation or heel bleeding on day 3 reached high prevalence values. Exudation at the tail base was also observed on day 3 in Farm 2. Differences between farms were not consistent across all days but varied depending on both observation day and lesion type (Table 6, Figure S1). Because the study included only two farms differing in several management, health, and housing characteristics, the present data do not allow conclusions regarding the specific causes of the observed between-farm differences. Lesion patterns differed clearly between body regions. At the tail base, swelling, exudation, and necrosis were predominant, whereas only low prevalences were observed at the tail tip. At the heels, swelling and bleeding were particularly frequent and reached high prevalences on specific days. Lesions affecting ears and coronary bands were less frequent overall but showed pronounced peaks in specific farm–day combinations (Table 6).

Sex effects were generally weak and inconsistent across lesion types, farms, and observation days. The only repeatedly observed and statistically significant sex difference concerned teat necrosis, which occurred more frequently in female piglets. Although isolated sex differences were detected for some individual lesion traits, no consistent pattern was evident across the overall SINS phenotype (Table 5 and Table 6).

In weaned piglets, prevalences were significantly lower, particularly for lesions at the tail base, coronary bands, heels, and teats, as well as for the overall SINS score (Table 7). In this age group, no significant effects of farm, age, or sex were detected. The prevalence of SINS signs was markedly lower than during the suckling period, and several lesion types were no longer observed, particularly on Farm 2 (Table S2).

In addition to the predefined SINS lesions, skin lesions at the olecranon and calcaneus were observed during the clinical examinations (Figure S2) and were prospectively recorded throughout the study. These lesions occurred in 19% of suckling piglets on both farms and were significantly associated with lesions at the coronary bands and other body regions (Table S3).

On Farm 1, increasing SINS scores—particularly heel bleeding, tail tip exudation, and teat swelling—were significantly associated with exudative lesions at the olecranon and/or calcaneus. In contrast, on Farm 2, associations were mainly observed with lesions of the coronary bands, especially redness and exudation (Table S3). These lesions were no longer present at weaning.

### 3.2. Hematology

Higher SINS scores in suckling piglets were associated with alterations in several hematological parameters measured at weaning (Table 8).

These associations included higher monocyte proportions and lower platelet counts. In addition, erythrocyte counts and hematocrit values were lower in piglets with higher SINS scores, whereas mean corpuscular volume (MCV) and mean corpuscular hemoglobin concentration (MCHC) were higher. Red cell distribution width (RDW) was also reduced.

Within the leukocyte compartment, lymphocyte counts and segmented neutrophils were associated with SINS scores. Given the exploratory nature of the analysis and the limited sample size, these findings should not be interpreted as evidence of a strictly monotonic dose–response relationship.

### 3.3. Tail Integrity in Finishing Pigs

At the beginning of the finishing period, only mild tail lesions were observed in 10.3% of pigs on Farm 1 and 1.6% on Farm 2. No associations were detected between these lesions and SINS signs observed during the suckling or weaning periods.

**Figure 5:**
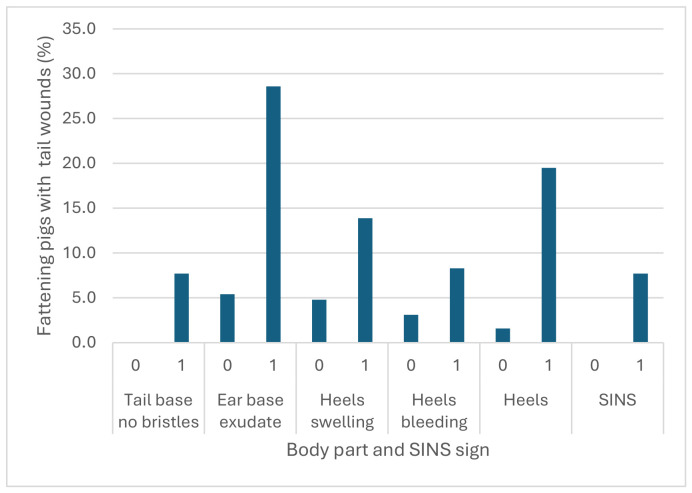
Associations between tail wounds in finishing pigs and SINS signs recorded during the suckling period. All associations were significant at *p* < 0.05. Data are shown exclusively for Farm 1, as no tail wounds were observed on Farm 2.

Ninety percent of finishers had intact tails. Slightly shortened but otherwise intact tails (score 2) were observed in seven pigs (six from Farm 1 and one from Farm 2). Tail wounds (score 4) occurred in 5.9% of pigs (*n* = 17), all from Farm 1. Overall, 79.8% of finishers had shown SINS signs as suckling piglets. Notably, all pigs with tail wounds at this phase (*n* = 17) had exhibited SINS as suckling piglets, whereas none of the pigs without SINS signs (*n* = 65) developed tail wounds. This association was statistically significant (*p* < 0.011, χ^2^ test), although the number of affected animals was low. The prevalence of tail wounds was also significantly associated with multiple SINS signs and the overall SINS score (Figure 5).

### 3.4. Tail Integrity in Slaughter Pigs

Overall, 76% of slaughter pigs had tail lengths > 30 cm, while 23% had tail lengths between 23 and 30 cm. Only one pig (Farm 1) had a tail length of 16–22 cm. Tails > 30 cm were observed in 74.2% of pigs on Farm 1 and 85.4% on Farm 2, with no significant difference between farms.

**Figure 6:**
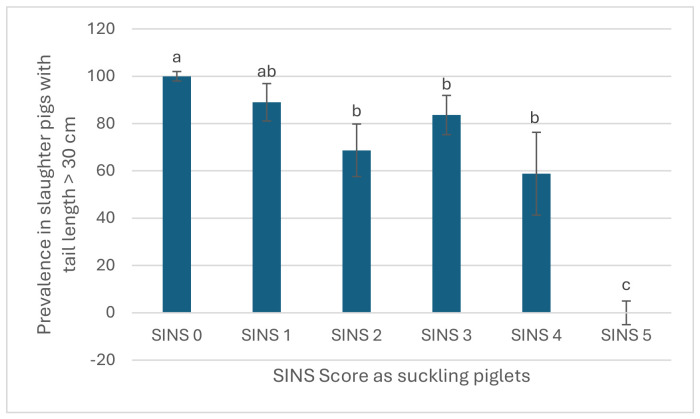
Prevalence of intact tails (>30 cm) in slaughter pigs by SINS score of the individuals as suckling piglets. Columns with different letters are statistically significantly different (*p* ≤ 0.05).

A significant effect of SINS score in suckling piglets on tail length at slaughter was detected (*p* < 0.001; Figure 6). The probability of an intact tail (>30 cm) decreased with increasing SINS score, ranging from 99.9% (score 0) to 0% (score 5). The numbers of pigs included in the respective SINS-score groups (scores 0–5) were 50, 62, 56, 51, 21, and 10 animals, respectively.

At slaughter, tails were free of abnormalities in 85% of pigs, while kinks, local size deviations, and healed wounds were observed in 12%, 1%, and 2% of pigs, respectively. No signs of ring formation, open wounds, infection, or necrosis were observed.

Pigs with tails > 30 cm showed significantly fewer abnormalities compared with pigs with shorter tails (<30 cm), including lower prevalence of kinks and healed wounds. No differences between farms were detected.

**Figure 7:**
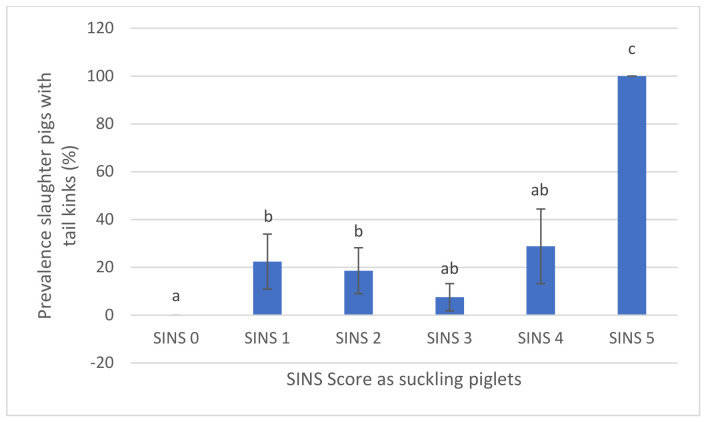
Prevalence of tail kinks at slaughter by SINS score of the individuals as suckling piglets. Columns with different letters are statistically significantly different (*p* ≤ 0.05).

**Figure 8:**
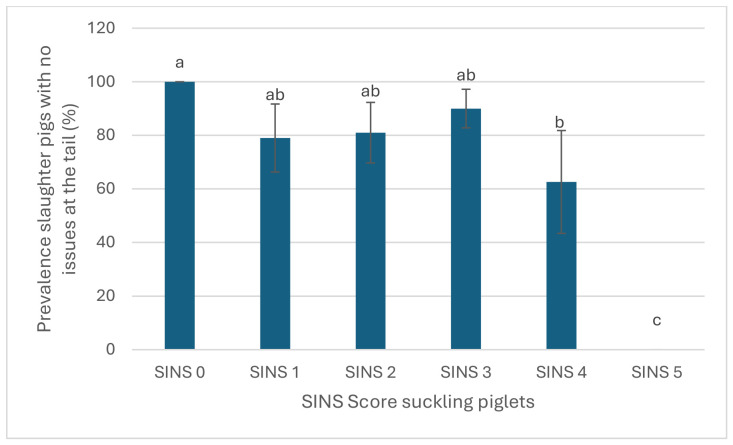
Prevalence of slaughter pigs with no tail issues by SINS score of the individuals as suckling piglets. Columns with different letters are statistically significantly different (*p* ≤ 0.05).

While no associations were found between SINS scores and healed wounds or size deviations, SINS scores were significantly associated with the occurrence of tail kinks (Figure 7) and the absence of any tail abnormalities (Figure 8).

### 3.5. Associations with Performance Traits

Sows on Farm 2 had significantly lower parity compared with those on Farm 1 (*p* < 0.001). Animals from Farm 2 showed lower weaning weights but higher slaughter weights, along with lower carcass weights, muscle thickness, and meat-to-fat ratio. However, none of these parameters were associated with SINS scores.

## 4. Discussion

### 4.1. SINS Prevalence in an International Context

Compared with previously published studies [10,39,40,46,47,48], the present study showed overall lower prevalences of SINS lesions, particularly in weaned piglets and in specific body regions such as the tail tip, ears, and coronary bands in suckling piglets. In contrast, prevalences at the tail base, teats, and heels were within the mid-range of previously reported values.

Similarly low prevalences have been described in pigs raised under specific production conditions in Northern Italy [9]. However, while results in weaned piglets were comparable, SINS manifestations in suckling piglets were more pronounced in the present study. These differences may be associated with variations in genetics, management, and production systems. In particular, slower-growing and less lean genetic lines, as used in the Italian system, have been associated with reduced SINS prevalence [9].

Importantly, previous studies have demonstrated substantial variability in SINS occurrence even within conventional production systems [9,10], indicating that low SINS prevalence is not restricted to specific production types but can also be achieved under optimized conditions.

The farms investigated in the present study were part of an organic production system characterized by lower stocking density, increased use of straw bedding, higher dietary fiber content, and generally more robust genetics. These factors have been proposed to improve gut health and may reduce systemic inflammatory stimuli. In addition, improved flooring conditions may reduce local mechanical stress and enhance tissue integrity.

At the same time, potential risk factors such as increased exposure to mycotoxins due to limited use of feed additives and increased contact with environmental materials should be considered. The net effect of these factors likely reflects a complex balance between protective and risk-modifying influences.

An additional factor potentially contributing to the low prevalence of SINS in weaned piglets is the extended suckling period. Piglets remained with the sow for at least six weeks, which may have reduced weaning-associated stress. Delayed weaning has been shown to lower physiological stress responses, including reduced plasma cortisol levels [49]. Since stress is known to activate systemic inflammatory pathways [50,51,52], the observed findings are compatible with the hypothesis that reduced stress exposure may be associated with lower SINS expression. Transcriptomic data further support a link between stress signaling and inflammatory processes in SINS [11,42,43].

Taken together, these findings suggest that management factors such as weaning age, housing conditions, and feeding strategies may substantially influence SINS expression, particularly in later production stages.

### 4.2. Endogenous Versus Mechanically Modulated SINS Lesions

The present findings are consistent with the hypothesis that SINS represents a systemic, endogenously influenced condition, with mechanical factors acting as modulators rather than primary causes of lesions [9,11,44,53,54].

Body regions in direct contact with the floor, such as heels, coronary bands, and joints, showed the greatest variation in lesion prevalence. However, the temporal development, morphology, and co-occurrence with other SINS signs may indicate that many of these lesions originate from systemic inflammatory processes.

SINS-associated lesions typically appear early in life (days 2–3) for the first time and are characterized by swelling, exudation, and necrosis, reflecting inflammatory and ischemic processes [55]. In contrast, purely mechanical lesions such as abrasions or superficial bleeding tend to occur later and increase with cumulative mechanical load [10].

The present data support an interaction model: early systemic tissue alterations increase tissue vulnerability, thereby amplifying the impact of mechanical stress. This is consistent with previous findings showing that early lesion patterns strongly predict later lesion development under mechanical load [10].

Examples from the current study illustrate this interaction. Heel swelling showed a clear temporal pattern consistent with systemic SINS processes and marked differences between farms, indicating an endogenous origin. In contrast, heel bleeding increased with age and likely represents mechanically induced damage that is amplified in SINS-affected tissue.

Similarly, coronary band lesions displayed distinct patterns. Redness appeared to be a non-specific or mechanically induced change, whereas exudation showed characteristics of a systemic inflammatory lesion with strong farm-specific modulation.

Tail tip lesions were consistently associated with tail base inflammation and showed a pattern consistent with secondary ischemic processes rather than direct mechanical injury. This interpretation is supported by previous perfusion studies demonstrating reduced blood flow in affected tissues [55].

Notably, exudative lesions at the elbow and hock joints were observed for the first time in association with SINS. Their early onset, strong association with other SINS signs, and pronounced farm specificity are consistent with the hypothesis that these lesions constitute an additional manifestation of the systemic inflammatory process rather than purely mechanical damage.

Overall, these findings support a model in which SINS represents a systemic inflammatory syndrome that predisposes tissues to secondary mechanical damage, particularly in floor-exposed regions.

### 4.3. Farm Effects and Modulating Factors

Differences in SINS severity between farms, as well as the overall comparatively low prevalence observed in the investigated organic herds, may partly be explained by differences in environmental conditions, management practices and resilience-promoting factors. Because multiple farm characteristics differed simultaneously, the present study does not allow identification of the specific factors responsible for the observed differences. Therefore, the following considerations should be regarded as possible explanations rather than causal interpretations of the observed farm effects. Several studies indicate that environmental enrichment, improved early-life conditions and reduced chronic stress can positively influence immune competence, health and behavioural stability in pigs [56,57,58]. Enriched housing conditions have been shown to reduce disease susceptibility, inflammatory responses and severity of co-infections in pigs [56,58], while repeated social stressors such as regrouping and mixing may impair resilience and increase vulnerability to health disturbances [59]. In addition, nutritional factors appear to influence inflammatory and behavioural disorders, as amino acid supplementation was shown to reduce tail biting more effectively than additional enrichment under low-protein feeding conditions [60]. Organic farming systems are often characterised by lower stocking density, more complex environments, increased exploratory opportunities, roughage access and reduced intensity of management interventions, all of which may contribute to improved gut integrity, lower chronic stress exposure and enhanced immunological stability [56,58]. Such factors may reduce systemic inflammatory activation and thereby contribute to the comparatively low SINS severity observed in the present study. At the same time, variation between farms suggests that even within organic systems, differences in management, nutrition, social stability and environmental complexity may substantially modulate the expression of SINS.

Farm 1 showed consistently higher prevalences across multiple lesion types and time points. These differences are likely multifactorial and may include variations in genetics, health status, management practices, and environmental conditions. In particular, genetic influences may play a role, as previous studies have demonstrated associations between SINS scores in breeding animals and their offspring [12,53,54,55].

Differences in weaning management may also contribute. On Farm 1, piglets were mixed immediately after weaning, whereas on Farm 2, piglets remained in the farrowing environment for a longer period before mixing. Such differences may influence stress exposure, social competition, and subsequent inflammatory responses and may therefore have contributed to differences in later tail outcomes between farms.

Health status differences, including the presence of enteric pathogens on Farm 1, may further contribute to systemic inflammatory load. However, no direct association with tail lesions in later life was observed. Overall, these findings emphasize that SINS expression is strongly modulated by farm-specific conditions, even under generally favorable production systems.

### 4.4. Systemic Effects: Hematological Associations

A key finding of the present study is the association between SINS severity in suckling piglets and systemic hematological changes observed at weaning. In agreement with previous studies [41], increased SINS severity was associated with elevated monocyte proportions and reduced platelet counts, indicating activation of inflammatory and coagulation pathways.

In addition, alterations in red blood cell parameters were observed for the first time. Decreased erythrocyte counts and hematocrit values, combined with increased MCV and MCHC, suggest the presence of a subclinical anemia consistent with inflammatory processes. These changes may be mediated by cytokine-induced alterations in iron metabolism and erythropoiesis, as described in inflammatory conditions [61,62].

The observed decrease in red cell distribution width (RDW) indicates a relatively uniform alteration of erythrocyte populations, which may reflect a coordinated systemic response rather than heterogeneous erythropoietic disturbances [63]. Furthermore, reductions in lymphocytes and segmented neutrophils were observed, consistent with stress- and inflammation-associated immune modulation [64,65]. These findings support the concept that SINS is not merely a local dermatological condition but involves systemic inflammatory processes that become measurable at later stages of development. Given the exploratory nature of the hematological analyses and the limited sample size, these associations should be interpreted cautiously and require confirmation in larger datasets.

### 4.5. Early-Life SINS and Tail Integrity

Although tail lesions were generally rare and mild in the studied herds, a clear association between early-life SINS and later tail integrity was identified.

All pigs exhibiting tail wounds at the beginning of the finishing period had previously shown SINS lesions as suckling piglets. Moreover, SINS severity was significantly associated with reduced tail length at slaughter, indicating that early inflammatory processes may lead to partial tissue loss independent of tail biting [9].

Since tail docking was not performed and no relevant tail biting was observed, these findings are consistent with the hypothesis that early-life inflammatory and necrotic processes may contribute to later tail integrity under the given conditions.

Tail length variation at slaughter may therefore reflect a combination of genetic factors and early-life inflammatory damage rather than solely behavioral causes such as tail biting [9].

These results highlight the importance of considering early-life health status when evaluating tail outcomes at slaughter and suggest that SINS may serve as a prognostic indicator for tail integrity [9,10].

### 4.6. Limitations of the Study

Several limitations should be considered when interpreting the present findings. First, the study was conducted on only two organic pig farms. Consequently, farm-specific effects cannot be disentangled from differences in management, health status, genetics, housing conditions, or biosecurity measures. The observed between-farm differences should therefore not be interpreted as evidence for causal effects of specific management factors.

Second, the study had an observational design and was not intended to identify causal mechanisms underlying SINS development or subsequent tail lesions. Proposed pathophysiological pathways discussed in the Introduction were not directly investigated.

Third, hematological analyses were based on a relatively small subset of animals (*n* = 35), resulting in limited numbers within some SINS-score categories. Therefore, the observed hematological associations should be regarded as exploratory and hypothesis-generating.

Fourth, because all farms operated under certified organic conditions, extrapolation of the findings to conventional production systems should be made with caution. Validation in larger studies including a greater number of farms and different production systems is warranted.

Finally, a large number of traits and associations were evaluated. Given the exploratory nature of the study, no formal adjustment for multiple testing was applied. Consequently, individual statistically significant findings should be interpreted with caution and emphasis should be placed on consistent patterns observed across traits and production stages.

## 5. Conclusions

The present study demonstrates that SINS is associated with systemic hematological alterations and subsequent tail outcomes under the investigated organic farming conditions. Although the study was limited to two farms and should be regarded as exploratory, the findings suggest that SINS scoring may have potential as an animal-based risk indicator for identifying pigs at increased risk of later tail damage. Further studies involving larger numbers of farms and different production systems are required to validate these findings and to determine the practical applicability of SINS scoring for welfare assessment and risk prediction.

## Figures and Tables

**Table 1:** Summary of major characteristics of the two organic pig farms.

Characteristic	Farm 1	Farm 2
Number of sows	99	62
Sow genetics	Topigs L × T	Topigs L × Z
Terminal boars	PIC	Tempo-growth
PRRS status	Unstable	Stable
PRRS vaccination piglets	Yes	No
Additional piglet vaccinations	PCV2, *M. hyopneumoniae*, PIA, *B. pilosicoli*	PCV2, *M. hyopneumoniae*, PIA
Nursery health problems	PIA observed	None observed
Nursery mortality	1.5–3.0%	<1.5%
Weaning age (days)	46.6 ± 4.2	50.8 ± 2.5
Mixing after weaning	Immediate mixing and transfer	Remained in farrowing unit for ~2 weeks
Nursery group size	20 pigs	10–15 pigs
Nursery flooring	Straw-bedded concrete floor and metal slats	Straw-bedded concrete floor
Nursery drinkers	5 bowl drinkers/pen	4 nipple drinkers/pen
Finisher group size	60 pigs	40 pigs
Finisher flooring	Straw-bedded concrete floor	Straw-bedded concrete floor with metal slats
Water supply in finishers	1 drinker per 10 pigs	1 nipple drinker per 5 pigs
Mycotoxin binder in feed	No	No
Outdoor access	Yes	Yes

**Table 2:** Neonatal and weaned piglets clinical assessment.

Body Part	SINS Sign	Description
Tail base	Hairless	Less than normal or no hair visible
	Redness	Redness of the skin is visible
	Swelling	Palpable subcutaneous oedema
	Exudate	Inflammatory liquid visible at the surface
	Necrosis	Necrotic tissue visible at the surface
	Bleeding	Blood is visible
Tail tip	Redness	Redness of the skin is visible
	Swelling	Palpable subcutaneous oedema
	Exudate	Inflammatory liquid is visible at the surface
	Necrosis	Necrotic tissue is visible at the surface
	Bleeding	Blood is visible
Ear basis	Hairless	Less than normal or no hair is visible
	Redness	Redness of the skin is visible
	Exudate	Inflammatory liquid visible at the surface
Ear tip	Redness	Redness of the skin is visible
Coronary band	Redness	Redness of the skin is visible
	Exudate	Inflammatory liquid is visible at the surface
	Swelling	Swollen tissue is visible
	Subcutaneous bleeding	Blood is visible
Teats	Redness	Redness of the teats is visible
	Swelling	Swollen tissue is visible
	Necrosis	Necrotic teats (black, dry) are visible
Heels	Swelling	Swollen tissue is visible
	Subcutaneous Bleeding	Blood is visible beneath the skin surface

**Table 3:** Finishing pigs’ tail assessment.

Score	Description
Score 1	tail long and intact, with curl, no lesions
Score 2	tail long and intact, but shorter, still with curl, no lesions
Score 3	tail is shorter, no curl, healed, no acute lesions
Score 4	tail long with acute lesion or visible wound
Score 5	tail is shorter, with acute lesion bite wound

**Table 4:** Tail integrity in slaughter pigs.

Tail Lesion Category	Description
No issues	
Kink/axis deviation	A deviation from the normal longitudinal shape of the tail: sideways deviation
Local size deviation	A deviation from the normal circumference of the tail: local thickening
Scars	Visible signs of healing process of the surface of the tail: uneven surface

**Table 5:** Prevalence of affected body parts and prevalence of SINS-positive suckling piglets.

	Farm 1	Farm 2	D ^1^ 1	D2	D3	D4	D5	D6	Male	Female
Tail base	44.1 ± 6.7	29.6 ± 4.8	21 ± 13.8	29.6 ± 11.5	91.6 ± 4.8	11.7 ± 6.7	39 ± 10.4	23.7 ± 13.7	35.2 ± 5.1	39.8 ± 6.8
Tail tip	11.9 ± 4.6	1 ± 1.3	7.6 ± 6.2	14.6 ± 8.3	2.1 ± 1.7	1.2 ± 1.5	0 ± 0	16.4 ± 10.9	6.7 ± 3.6	7.1 ± 3.7
Ear	7 ± 2	0 ± 1.9	0 ± 2.2	0 ± 2.1	3.3 ± 2.8	0 ± 3	4.5 ± 2.4	13.3 ± 5.5	4.9 ± 1.9	5.4 ± 2
Coronary bands	0.3 ± 0.4 a	29.2 ± 5.6 b	0 ± 0	6.4 ± 5.5	46.4 ± 5.8	2.3 ± 3.9	7.6 ± 5.9	11.2 ± 9	11.1 ± 3.2	16.4 ± 7.7
Heels	68.4 ± 5.4 a	42.1 ± 9.3 b	23.2 ± 14.6	28.8 ± 15.2	86.4 ± 8.3	49.6 ± 5.6	70.4 ± 14.2	63.7 ± 14.7	52.5 ± 6.8	52.7 ± 6.8
Teats	40.6 ± 4.7	39.3 ± 7.8	23.1 ± 8.6	37.8 ± 8.4	52 ± 12.7	35.2 ± 11.2	55.9 ± 8.5	27.6 ± 11.2	27.3 ± 5.4 a	52.7 ± 6.8 b
SINS	86.6 ± 4.5	70.7 ± 10.4	59 ± 19.1	76.6 ± 12.6	98.2 ± 1.9	62.4 ± 16.9	88.9 ± 9.5	81 ± 15	76.9 ± 7.6	81.8 ± 7.4

^1^ D: day of life; a, b: values with different letters within one category are statistically significant at *p* ≤ 0.05.

**Table 6:** Farm-effects by SINS sign, age and sex in suckling piglets.

Body Part	Sign	Farm	D ^1^ 1	D2	D3	D4	D5	D6	Male	Female
Tail Base	No bristles	Farm 1	25.8 ± 21.4	15.7 ± 13.7	59.7 ± 21.9 A	3.9 ± 4.7	6.8 ± 8.8	0 ± 0	19.7 ± 8.7	17.6 ± 7.7
		Farm 2	n.a.	12.5 ± 15	0 ± 0 B	0 ± 0	4.4 ± 6.3	5.3 ± 10	3.2 ± 4.5	4.5 ± 6.2
Tail Base	Swelling	Farm 1	4.5 ± 3.9	14.4 ± 9.5	68.9 ± 12.9 aA	26.7 ± 13.8	57.6 ± 17 aA	44 ± 20.6	33.6 ± 7.6	38.4 ± 8.5
		Farm 2	n.a.	6.1 ± 4.9	0 ± 4.9 B	0 ± 5.4	5.1 ± 4.0 B	4.4 ± 4.1	5.7 ± 2.9	4.3 ± 3
Tail Base	Exudation	Farm 1	0 ± 2.2	0 ± 2.7	6.4 ± 3.4 A	0 ± 2.7	5.1 ± 3.8	0 ± 4.1	4.1 ± 2	3.5 ± 1.7
		Farm 2	n.a.	4.9 ± 4.1	97.1 ± 4.9 B	0 ± 5.4	14.4 ± 6.4	4.4 ± 4.1	23.2 ± 2.5	24.7 ± 3.7
Tail Base	Necrosis	Farm 1	0 ± 2.2	4.7 ± 3.4	7.4 ± 3.8	0 ± 2.7	5.1 ± 3.8	7.1 ± 6	5.4 ± 2.3	4.6 ± 2.2
		Farm 2	n.a.	0	0	0	0	0	0	0
Tail Base	Bleeding	Farm 1	7.4 ± 3.4	10.4 ± 4.8	7.7 ± 3.7	0 ± 2.7	0 ± 2.8	11.9 ± 7.9	6.5 ± 2.7	7.8 ± 2.6
		Farm 2	n.a.	0	0	0	0	0	0	0
Tail tip	Redness	Farm 1	7.4 ± 3.4	10.4 ± 4.8	7.7 ± 3.7	0 ± 2.7	0 ± 2.8	11.9 ± 7.9	6.5 ± 2.7	7.8 ± 2.6
		Farm 2	n.a.	7.7 ± 4.8	0 ± 4.9	0 ± 5.4	0 ± 3.1	0 ± 3.6	0 ± 2.2	3.8 ± 3.3
Tail tip	Exudation	Farm 1	0 ± 2.2	0 ± 2.7	0 ± 2.4	0 ± 2.7	0 ± 2.8	7.7 ± 6.5	4.5 ± 2.4	0 ± 1.6
		Farm 2	n.a.	7.7 ± 4.8	0 ± 4.9	0 ± 5.4	0 ± 3.1	0 ± 3.6	0 ± 2.2	3.8 ± 3.3
Tail tip	Necrosis	Farm 1	0 ± 2.2	0 ± 2.7	4.4 ± 3	4.5 ± 3.4	0 ± 2.8	7.7 ± 6.5	5.6 ± 2.6	0 ± 1.6
		Farm 2	n.a.	7.7 ± 4.8	0 ± 4.9	0 ± 5.4	0 ± 3.1	0 ± 3.6	0 ± 2.2	3.8 ± 3.3
Tail tip	Bleeding	Farm 1	0 ± 2.2	4.4 ± 3.2	3.8 ± 2.6	0 ± 2.7	0 ± 2.8	0 ± 4.1	0 ± 1.7	3.7 ± 1.7
		Farm 2	n.a.	0	0	0	0	0	0	0
Ear	Exudation	Farm 1	0 ± 2.2 a	0 ± 2.7 a	3.8 ± 2.6 a	0 ± 2.7 a	6.1 ± 3.8	23.7 ± 10.3 bA	6.6 ± 2.9	7.5 ± 2.8
		Farm 2	n.a.	0	0	0	0	0 B	0	0
Coronary bands	Redness	Farm 1	0 ± 2.2	0 ± 2.7	0 ± 2.4	0 ± 2.7	4.3 ± 3.3	0 ± 4.1	0 ± 1.7	3.3 ± 1.7
		Farm 2	n.a.	12.5 ± 14.1	0 ± 0	0 ± 0	6.5 ± 8.9	18.9 ± 21.2	4.4 ± 4.7	7.6 ± 9.7
Coronary bands	Exudation	Farm 1	0	0	0 B	0	0	0	0	0
		Farm 2	n.a.	12.7 ± 11 b	90 ± 11.7 a	4.5 ± 7.9 bB	13.4 ± 11.7 b	21 ± 17.5 b	23.6 ± 6.8	28.9 ± 8.7
Heels	Swelling	Farm 1	2 ± 2.3	4 ± 4.9	55.1 ± 18.7 a	54.2 ± 19.8 aA	7.9 ± 11	0 ± 0	28.3 ± 6.9 a	12.8 ± 7.2 b
		Farm 2	n.a.	0	0 b	0 b	0	0	0	0
Heels	Bleeding	Farm 1	21.7 ± 10.8 a	25.4 ± 13.8 a	63 ± 14.7	94.2 ± 5.1 bA	96.7 ± 3.9 b	93.7 ± 10.1 A	62.1 ± 6.2	69.4 ± 5.9
		Farm 2	n.a.	28.1 ± 32.5	100 ± 0.1 a	4.5 ± 13.2 bB	43.3 ± 39	31.6 ± 34.7 B	37.1 ± 15.6	41.5 ± 19.7
Teats	Redness	Farm 1	5.4 ± 5	7.3 ± 7.7	2.1 ± 2.5	0 ± 0	0 ± 0	0 ± 0	2.3 ± 2	2.6 ± 2.4
		Farm 2	n.a.	9.3 ± 15.1	0 ± 0	0 ± 0	3.6 ± 5.6	7 ± 12.6	3.6 ± 5.3	4 ± 6.2
Teats	Swelling	Farm 1	0 ± 0	0 ± 0	0 ± 0	13.4 ± 26.4	14.2 ± 27.7	0 ± 0	0 ± 0	9.2 ± 12.8
		Farm 2	n.a.	0	0	0	0	0	0	0
Teats	Necrosis	Farm 1	3.1 ± 3.2 a	2.8 ± 3.7 a	67.8 ± 19 b	51.4 ± 18.2	82.8 ± 14.2 bA	44.5 ± 24.5	30.6 ± 10 a	53.5 ± 8.3 b
		Farm 2	n.a.	42.7 ± 13.9	55.2 ± 19.9	28.8 ± 19.5	28 ± 11.3 B	20 ± 12.5	24.5 ± 8.2 a	35.5 ± 11.6 b

^1^ D: day of life; a, b: values with different letters within one category are statistically significant at *p* ≤ 0.05. A, B: values with different capital letters are significantly different between farms. n.a.: values at day 1 were not available on farm 2.

**Table 7:** Prevalence of SINS signs in weaners by farm, age at scoring, and sex.

		Farm 1	Farm 2	D ^1^ 40	D45	D48	D52	Male	Female
Single signs									
Tail base	No bristles	7.1 ± 4.3	3 ± 4.8	3.1 ± 4.9	6.9 ± 6.4	0.6 ± 0.7	9.8 ± 8.8	4.2 ± 3.8	6.5 ± 5.1
	Exudate	3.6 ± 1.3	0 ± 3	0 ± 3.1	3.9 ± 4	3.2 ± 2.1	0 ± 1.7	3.1 ± 1.9	3.5 ± 2.2
Tail tip	No bristles	6.6 ± 1.8	0 ± 3	4.8 ± 3.8	0 ± 4	5 ± 2.3	7.3 ± 2.7	5.1 ± 2.2	4.9 ± 2.4
	Redness	3.6 ± 1.3	0 ± 3	0 ± 3.1	3.8 ± 4	3.3 ± 2.1	0 ± 1.7	3.2 ± 1.9	3.4 ± 2.2
	Exudate	3.1 ± 1.2	0 ± 3	0 ± 3.1	3.3 ± 4	0 ± 2.1	0 ± 1.7	0 ± 1.9	3.1 ± 2.2
	Necrosis	3 ± 1.2	0 ± 3	0 ± 3.1	0 ± 4	3.1 ± 2.1	0 ± 1.7	0 ± 1.9	3 ± 2.2
Ear	Exudate	3 ± 1.2	3.8 ± 3.1	0 ± 3.1	0 ± 4	3.1 ± 2.1	4.2 ± 2.1	3.2 ± 1.9	3.4 ± 2.3
Teats	Redness	3.8 ± 1.3	0 ± 3	0 ± 3.1	4.4 ± 4.1	3.2 ± 2.1	0 ± 1.7	3.3 ± 1.9	3.5 ± 2.2
Affected body parts									
Tail base	0/1	18.2 ± 5.6	20.3 ± 11.1	4.8 ± 5.8	24.5 ± 13	9.3 ± 7.5	30.8 ± 12.9	23.4 ± 9.7	14.8 ± 6
Tail tip	0/1	7.4 ± 1.8	0 ± 3	4.8 ± 3.8	4.4 ± 4.1	5 ± 2.3	7.3 ± 2.7	5.4 ± 2.2	5.5 ± 2.4
Ear	0/1	3 ± 1.2	3.8 ± 3.1	0 ± 3.1	0 ± 4	3.1 ± 2.1	4.2 ± 2.1	3.2 ± 1.9	3.4 ± 2.3
Teats	0/1	3.8 ± 1.3	0 ± 3	0 ± 3.1	4.4 ± 4.1	3.2 ± 2.1	0 ± 1.7	3.3 ± 1.9	3.5 ± 2.2
SINS	0/1	25.9 ± 4.5	21.8 ± 8.1	8.9 ± 6.6 a	29.5 ± 10.1	13.8 ± 5.9 a	36.7 ± 9 b	28.3 ± 7.1	20 ± 4.9

^1^ D: day of life; a, b: values with different letters within one category are statistically significant at *p* ≤ 0.05; 0/1: sign visible/not visible.

**Table 8:** Associations between hematological parameters in weaners and SINS-scores in suckling piglets.

Parameter	SINS 0 (*n* = 8)	SINS 1 (*n* = 4)	SINS 2 (*n* = 5)	SINS 3 (*n* = 11)	SINS 4 (*n* = 5)	SINS 5 (*n* = 2)	*p*
Monocytes (%)	4.7 ± 0.4 a	6 ± 0.5 b	5.6 ± 0.6 b	5.9 ± 0.4 b	6.5 ± 0.5 b	7.5 ± 1.3 b	0.031
Thrombocytes (K/µL)	545.7 ± 28.8 a	645.6 ± 36.9 b	476.4 ± 41.3 a	507.9 ± 27.7 a	548.2 ± 38 a	413.9 ± 94 a	0.014
Monocytes (K/µL)	1.37 ± 0.13	1.46 ± 0.17	1.42 ± 0.19	1.45 ± 0.13	1.69 ± 0.18	1.78 ± 0.43	n.s.
Lymphocytes (K/µL)	15.3 ± 0.7 a	12.4 ± 0.9 b	13.9 ± 1 ab	12.8 ± 0.7 b	13.2 ± 0.9 b	10.8 ± 2.3 ab	0.033
Segmented neutrophiles (K/µL)	12.4 ± 0.8 a	10.5 ± 1	10 ± 1.1 b	9.6 ± 0.7 b	10.6 ± 1	10.7 ± 2.5	0.161
RDW (%)	24.7 ± 0.4 a	24.9 ± 0.5 a	23.7 ± 0.6 ab	23.7 ± 0.4 ab	23.1 ± 0.5 b	22.6 ± 1.3 ab	0.029
MCHC (g/dL)	27.6 ± 0.8 acd	28.5 ± 0.8 abd	28.6 ± 0.8 c	28.5 ± 0.7 c	27.5 ± 0.8 d	29.2 ± 1.1 abcd	0.044
MCV (fL)	57.9 ± 0.9 b	55.4 ± 1.1 a	58.5 ± 1.3 b	60 ± 0.9 b	61.3 ± 1.2 b	56 ± 2.9 abcd	0.003
Htkt (%)	42.1 ± 1.1 a	39.3 ± 1.4 ab	40.8 ± 1.5 ab	37.2 ± 1 b	39.8 ± 1.4 ab	34.8 ± 3.5 b	0.031
Erythrocytes (M/µL)	7.25 ± 0.14 ac	7.08 ± 0.18 c	6.99 ± 0.2 c	6.23 ± 0.13 b	6.5 ± 0.18 b	6.22 ± 0.46 b	<0.001

MCV: mean corpuscular volume; MCHC: mean corpuscular hemoglobin concentration; Htkt: hematocrit; RDW: red cell distribution width (RDW); *p*: significance; n.s.: not significant. Values in a row with different letters are statistically significant (*p* ≤ 0.05).

## Data Availability

The data presented in this study are available upon reasonable request from the corresponding author. Restrictions apply to the availability of these data because they contain confidential farm-level information from participating commercial livestock enterprises.

## Supplementary Materials

The following supporting information can be downloaded at: https://www.mdpi.com/article/10.3390/ani16131962/s1, Table S1. Prevalences of SINS signs in suckling piglets by farm, age at scoring, and sex; Figure S1. Compilation of traits with significant farm effects in suckling piglets. Day: day of life; Table S2. Farm-Effects by SINS sign, age and sex in weaned piglets; Table S3. Significant associations between prevalence of exudated calcaneus and typical SINS signs by farm; Figure S2. Additional signs of inflammation and necrosis at the olecranon and calcaneus (Foto: Koenders-van Gog).

## Abbreviations

The following abbreviations are used in this manuscript:

D	Day (of life)
SINS	Swine Inflammation and Necrosis Syndrome
MAMPs	Microbe-associated molecular patterns
PCR	Polymerase chain reaction
MCHC	Mean corpuscular hemoglobin concentration
MCV	Mean corpuscular volume
RDW	Red cell distribution width
Htkt	Hematocrit
K/µL	Kilo/µL
M/µL	Million/µL
P	Significance
