# Supplementary material for: Early-Life Swine Inflammation and Necrosis Syndrome Is Associated with Later Tail Integrity and Systemic Hematological Changes in Organically Raised Pigs
Source: Animals (Basel). 2026 Jun 25;16(13):1962. doi: 10.3390/ani16131962 (PMC13359522; doi:10.3390/ani16131962)
Supplement: Supplementary file 1 [file animals-16-01962-s001.zip › animals-4370100-supplementary.pdf]

Supplementary Data

Early-Life Swine Inflammation and Necrosis Syndrome Is Associated with Later Tail Integrity and Systemic Hematological Changes in Organically Raised Pigs

Karien Koenders-van Gog, Esther Krooshoop, Thomas Wijnands and Gerald Reiner

Table S1. Prevalences of SINS signs in suckling piglets by farm, age at scoring and sex.

		Farm 1	Farm 2	P_{Farm}	D1	D2	D3	D4	D5	D6	P_{Day}	Male	Female	P_{Sex}
Tail base	No bristles	17.7 ± 5.7	5.5 ± 4.5		22.1 ± 19.7	15.6 ± 10.8	28.7 ± 11	1.9 ± 2.3	5.9 ± 5.6	3.7 ± 6.8		11.9 ± 5.2	12.4 ± 5.3	
	Redness	6.9 ± 1.8	4.2 ± 2.1		5.8 ± 3	7.7 ± 3.2	5.7 ± 3.2	0 ± 3	7 ± 3.2	5 ± 3.5		5.3 ± 1.7	6 ± 2	
	Swelling	35.1 ± 5.4a	2.1 ± 1.6b	*	4.8 ± 3.9a	9.9 ± 5.5a	33.7 ± 6.1b	12.6 ± 6.3a	29.4 ± 8.3ab	22.3 ± 10.1ab	*	19.8 ± 4.2	20.4 ± 4.4	
	Exudation	3.9 ± 1.3	24.7 ± 2.3	*	0 ± 2.1a	3.9 ± 2.4a	51.8 ± 3.0b	0 ± 3.0a	9.8 ± 3.7a	3.6 ± 2.9a	*	12.8 ± 1.6	13.9 ± 1.9	
	Necrosis	5 ± 1.6	0 ± 1.9		0 ± 2.2	3.8 ± 2.4	5.1 ± 3.1	0 ± 3	4 ± 2.5	5 ± 3.5		4.3 ± 1.6	3.8 ± 1.8	
Tail tip	Redness	7.2 ± 1.9	3.8 ± 2		7.4 ± 3.4	9 ± 3.4	5.3 ± 3.1	0 ± 3	0 ± 2.1	7.4 ± 4.3		4.9 ± 1.8	6.5 ± 2.1	
	Exudation	3.7 ± 1.4	0 ± 1.9		0 ± 2.1	0 ± 2.1	0 ± 2.7	0 ± 3	0 ± 2.1	5.3 ± 3.7		3.8 ± 1.7	0 ± 1.6	
	Necrosis	4.2 ± 1.5	0 ± 1.9		0 ± 2.2	0 ± 2.1	3.7 ± 2.9	3.7 ± 3.2	0 ± 2.1	5.3 ± 3.7		4.3 ± 1.7	0 ± 1.6	
Earbase	Exudation	7 ± 2	0 ± 1.9		0 ± 2.2	0 ± 2.1	3.3 ± 2.8	0 ± 3	4.5 ± 2.4	13.3 ± 5.5		4.9 ± 1.9	5.4 ± 2	
Coronary bands	Redness	0.3 ± 0.5	8 ± 5		0 ± 0	6.3 ± 6.4	0 ± 0	0 ± 0.1	4.4 ± 4.5	10.1 ± 9.9		2.1 ± 2	5.4 ± 4.1	
	Exudation	0 ± 0	28.9 ± 5.5	*	0 ± 0a	6.4 ± 5.5a	46.4 ± 5.9b	2.3 ± 3.9a	6.7 ± 5.8a	10.5 ± 8.7a	*	10.7 ± 3.1	15.5 ± 4	
Heels	Swelling	20.5 ± 6	0 ± 1	*	2 ± 2.3	2 ± 2.6	27.6 ± 9.4	27.1 ± 10	4 ± 5.8	0 ± 2		15.4 ± 4.2	7 ± 4.4	
	Bleeding	65.7 ± 5.6	42.3 ± 12.6	*	20.4 ± 12.7	26.5 ± 14	82.2 ± 17.7	49.5 ± 7.1	70.4 ± 13.6	63.9 ± 18.7		51.2 ± 7.2	58.8 ± 10	
Teats	Redness	2.3 ± 1.6	4.5 ± 3.9		5 ± 5	8.6 ± 8	1 ± 1.2	0 ± 0	2 ± 2.6	4.2 ± 6.2		3.1 ± 2.6	3.5 ± 2.9	
	Swelling	4.6 ± 6.4	0 ± 0		0 ± 0	0 ± 0	0 ± 0	6.7 ± 13.2	7.1 ± 13.9	0 ± 0		0 ± 0	5 ± 7	
	Necrosis	41.4 ± 5.9	37.4 ± 11.1		3.4 ± 3.2	22.7 ± 12.8	64.7 ± 16.6	43 ± 17	54.9 ± 11.9	30.7 ± 14.5		28.4 ± 7.8a	50.8 ± 9.0b	*

D: day of live; P: significance; *: the effect is significant for this parameter; a, b: values with different letters within one category are statistically significant at $P \leq 0.05$.

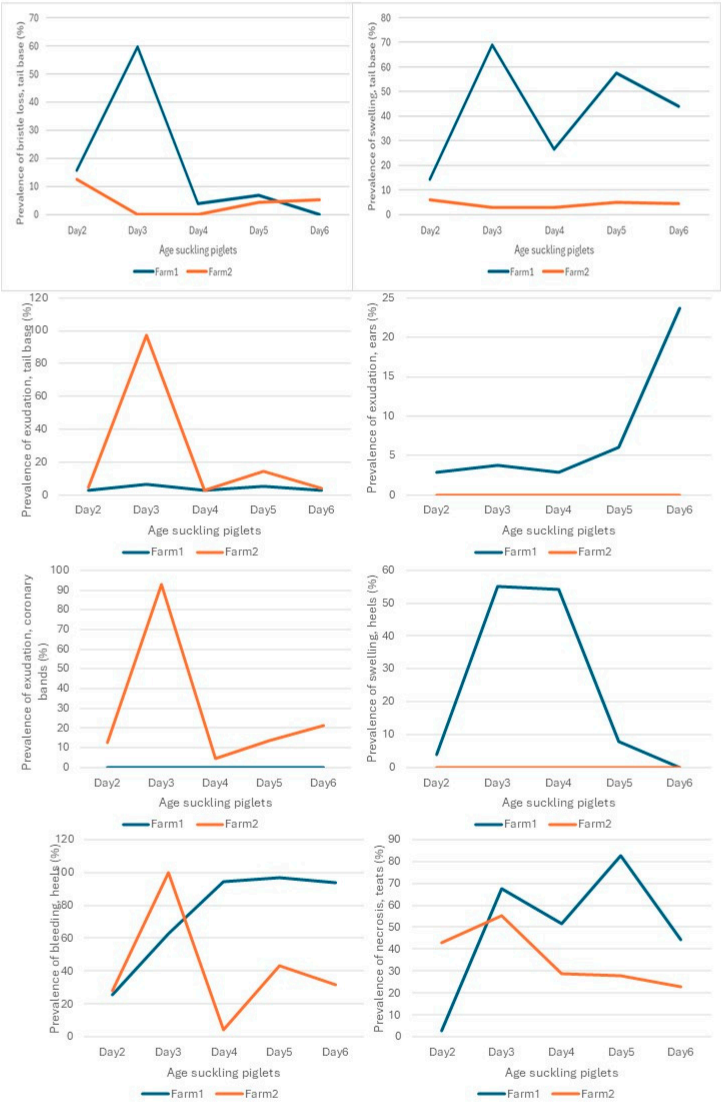

Figure S1. Compilation of traits with significant farm effects in suckling piglets. Day: day of live.

Table S2. Farm-Effects by SINS sign, age and sex in weaned piglets.

			D40	D45	D48	D50	Male	Female
Tail base	No bristles	Farm1	3.2 ± 5	13.8 ± 12.5	1.2 ± 1.3	11.4 ± 10.9	7.9 ± 5.6	5.3 ± 6.7
		Farm2	n.a.	0 ± 7.8	0 ± 4.2	9.1 ± 6.7	4.9 ± 4.6	0 ± 5.8
Tail base	Exudate	Farm1	0 ± 3.1	4.9 ± 2.7	3.5 ± 1.9	0 ± 2.5	5.1 ± 1.9	3.9 ± 1.8
		Farm2	n.a.	0 ± 7.7	0 ± 4	5.5 ± 4.1	3.1 ± 4	0 ± 5
Tail tip	No bristles	Farm1	3.4 ± 3.7	0 ± 0	5.2 ± 2.2	7.9 ± 4.3	0 ± 2	0 ± 2.3
		Farm2	n.a.	0	0	0	0	0
Tail tip	Redness	Farm1	0 ± 3.1	4.8 ± 2.7	3.8 ± 1.8	0 ± 2.5	0 ± 1.9	0 ± 1.7
		Farm2	n.a.	0	0	0	0	0
Tail tip	Exudate	Farm1	0 ± 3.1	3.8 ± 2.4	0 ± 1.7	0 ± 2.5	0 ± 1.8	0 ± 1.7
		Farm2	n.a.	0	0	0	0	0
Tail tip	Necrosis	Farm1	0 ± 3.1	0 ± 2.1	3.3 ± 1.7	0 ± 2.5	0 ± 1.8	0 ± 1.6
		Farm2	n.a.	0	0	0	0	0
Teats	Redness	Farm1	0 ± 3.1	5.9 ± 3	3.5 ± 1.9	0 ± 2.5	0 ± 1.9	0 ± 1.8
		Farm2	n.a.	0	0	0	0	0
Tail base	Any	Farm1	4.8 ± 5.8	32.3 ± 14.6	5.2 ± 3.4	30.6 ± 15.5	51.9 ± 7.6	9.1 ± 8.1
		Farm2	n.a.	18.1 ± 23.2	13.4 ± 15.3	31 ± 22	18.7 ± 21.2	23 ± 10.4
Tail tip	Any	Farm1	3.4 ± 3.5	4.9 ± 3	5.7 ± 2.4	8.7 ± 4.5	0 ± 2.3	0 ± 2.6
		Farm2	n.a.	0	0	0	0	0
Ears	Any	Farm1	0 ± 3.1	0 ± 2.1	3.3 ± 1.7	0 ± 2.5	5.1 ± 1.8	3.9 ± 1.6
		Farm2	n.a.	0 ± 7.7	0 ± 4	5.5 ± 4.1	0 ± 4	0 ± 5
Teats	Any	Farm1	0 ± 3.1	5.9 ± 3	3.5 ± 1.9	0 ± 2.5	0 ± 1.9	0 ± 1.8
		Farm2	n.a.	0	0	0	0	0
SINS		Farm1	8.9 ± 6.6a	42.5 ± 11.2b	14.1 ± 5.2a	38.2 ± 11.5b	55.6 ± 6.2	10.1 ± 6.6
		Farm2	n.a.	17 ± 23	13.2 ± 15.5	35.7 ± 23.9	23.8 ± 21.6	23 ± 11.3

Table S3. Significant associations between prevalence of exudated calcaneus and typical SINS signs by farm.

	Farm 1				Farm 2			
Score	0	1	2	P	0	1	2	P
Heels	12.1	18	33.3	0.24				
Tail tip Exudation	17.3	100	0	0.032				
Teats swelling	16.4	62.5	0	0.005				
Heels bleeding	11.1	22.4	0	0.016				
Coronary bands					17.9	21.4	50	0.019
Coronary bands exudation					17.6	37.9	0	0.026
Coronary bands redness					18.6	47.1	0	0.015

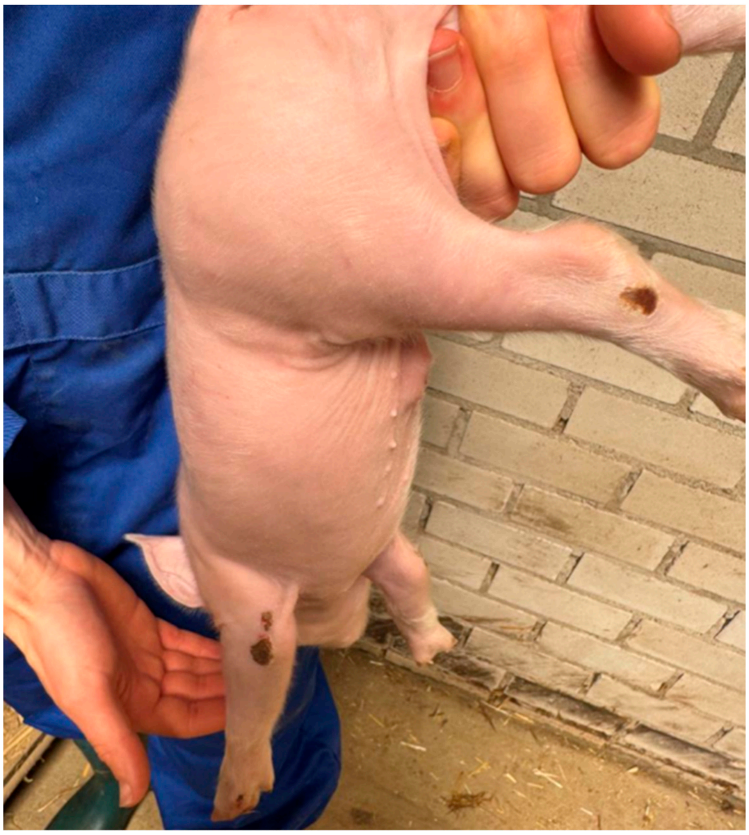

Figure S2. Additional signs of inflammation and necrosis at the olecranon and calcaneus (Foto: Koenders-van Gog)
